# Induction of Premature Senescence by Hsp90 Inhibition in Small Cell Lung Cancer

**DOI:** 10.1371/journal.pone.0011076

**Published:** 2010-06-11

**Authors:** Ian J. Restall, Ian A. J. Lorimer

**Affiliations:** 1 Centre for Cancer Therapeutics, Ottawa Hospital Research Institute, Ottawa, Ontario, Canada; 2 Department of Biochemistry, Microbiology and Immunology, University of Ottawa, Ottawa, Ontario, Canada; 3 Department of Medicine, University of Ottawa, Ottawa, Ontario, Canada; Roswell Park Cancer Institute, United States of America

## Abstract

**Background:**

The molecular chaperone Hsp90 is a promising new target in cancer therapy and selective Hsp90 inhibitors are currently in clinical trials. Previously these inhibitors have been reported to induce either cell cycle arrest or cell death in cancer cells. Whether the cell cycle arrest is reversible or irreversible has not generally been assessed. Here we have examined in detail the cell cycle arrest and cell death responses of human small cell lung cancer cell lines to Hsp90 inhibition.

**Methodology/Principal Findings:**

In MTT assays, small cell lung cancer cells showed a biphasic response to the Hsp90 inhibitors geldanamycin and radicicol, with low concentrations causing proliferation arrest and high concentrations causing cell death. Assessment of Hsp90 intracellular activity using loss of client protein expression showed that geldanamycin concentrations that inhibited Hsp90 correlated closely with those causing proliferation arrest but not cell death. The proliferation arrest induced by low concentrations of geldanamycin was not reversed for a period of over thirty days following drug removal and showed features of senescence. Rare populations of variant small cell lung cancer cells could be isolated that had additional genetic alterations and no longer underwent irreversible proliferation arrest in response to Hsp90 inhibitors.

**Conclusions/Significance:**

We conclude that: (1) Hsp90 inhibition primarily induces premature senescence, rather than cell death, in small cell lung cancer cells; (2) small cell lung cancer cells can bypass this senescence through further genetic alterations; (3) Hsp90 inhibitor-induced cell death in small cell lung cancer cells is due to inhibition of a target other than cytosolic Hsp90. These results have implications with regard to how these inhibitors will behave in clinical trials and for the design of future inhibitors in this class.

## Introduction

Hsp90 functions as a chaperone in normal cells, promoting the correct folding of both newly synthesized proteins and proteins that have been partially denatured due to stress [1]. It appears to be primarily involved in late stages of folding, probably by recognizing exposed hydrophobic surfaces on partially folded proteins. The basic mechanism of Hsp90-induced protein folding involves conformational switching between open and closed conformations that is regulated by ATP hydrolysis [2]. Rates of Hsp90 ATP hydrolysis are controlled in turn by its association with various cochaperones.

Although the number of proteins known to require Hsp90 for correct folding continues to increase, Hsp90 is clearly selective for a subset of cellular proteins. These include a number of proteins with known oncogenic activity, including Her2, Raf1 and Cdk4 [3]. In some cases Hsp90 shows preferential association with the mutant, oncogenic forms of proteins; this has been shown for both Src kinase and the EGF receptor [4]-[6]. Hsp90 also shows an increased association with cochaperones and higher ATPase activity in cancer cells, both *in vitro* and *in vivo* [7]. For these reasons there is considerable interest in Hsp90 as a target for cancer therapy.

Geldanamycin and radicicol are two structurally unrelated natural products that bind to the ATP binding site of Hsp90, blocking the conformational cycling that is necessary for its chaperone activity. These compounds show good selectivity for Hsp90, although they also bind to the Hsp90 endoplasmic reticulum paralog Grp94 and the Hsp90 mitochondrial paralog Trap1 at higher concentrations [8]-[10]. While these compounds establish in principle that Hsp90 is a “druggable” target from a pharmacology perspective, poor solubility and non-specific toxicities make them unsuitable for use in humans. Derivatized versions of geldanamycin have been produced that have improved pharmacological properties, although they still have some of the limitations of the parent compound [11]. In spite of this, there is evidence from some trials that Hsp90 inhibition is achievable, based on biomarker analysis in patient lymphocytes [12], [13] and tumour samples [14]. There is also some evidence for anticancer activity [15]. Recently novel Hsp90 inhibitors have been developed that do not have the limitations of previous compounds, and these are now entering clinical trials [11]. With these advances in the pharmacology of Hsp90 inhibition, a critical new area of investigation will be the identification of subsets of cancer patients that are most likely to benefit from Hsp90 inhibition.

Lung cancer is the largest cause of cancer deaths worldwide. About 15% of lung cancers are of a subtype known as small cell lung cancer. This cancer usually presents as metastatic disease and is usually not treated with surgery. Small cell lung cancer generally responds very well to radiation and chemotherapy initially, but the majority of patients relapse with resistant disease and die within two years [16]. The majority of small cell lung cancers have neuroendocrine properties and actively secrete polypeptide hormones [17]. These secreted hormones cause a range of paraneoplastic syndromes that are common complications of small cell lung cancer.

Here we have investigated the response of small cell lung cancer cells to Hsp90 inhibition. A previous study had shown that Hsp90 inhibitors induce cell death in small cell lung cancer cells via activation of the intrinsic pathway of apoptosis [18]. Our findings are consistent with this, but we observed that cell death only occurs at concentrations far higher than those required for inhibition of cytosolic Hsp90. We observe that treatment of small cell lung cancer cells with Hsp90 inhibitors at concentrations that are sufficient to inhibit cytosolic Hsp90 induces premature senescence rather than cell death.

## Results

### Response of human small cell lung cancer cell lines to Hsp90 inhibition

**Figure 1:**
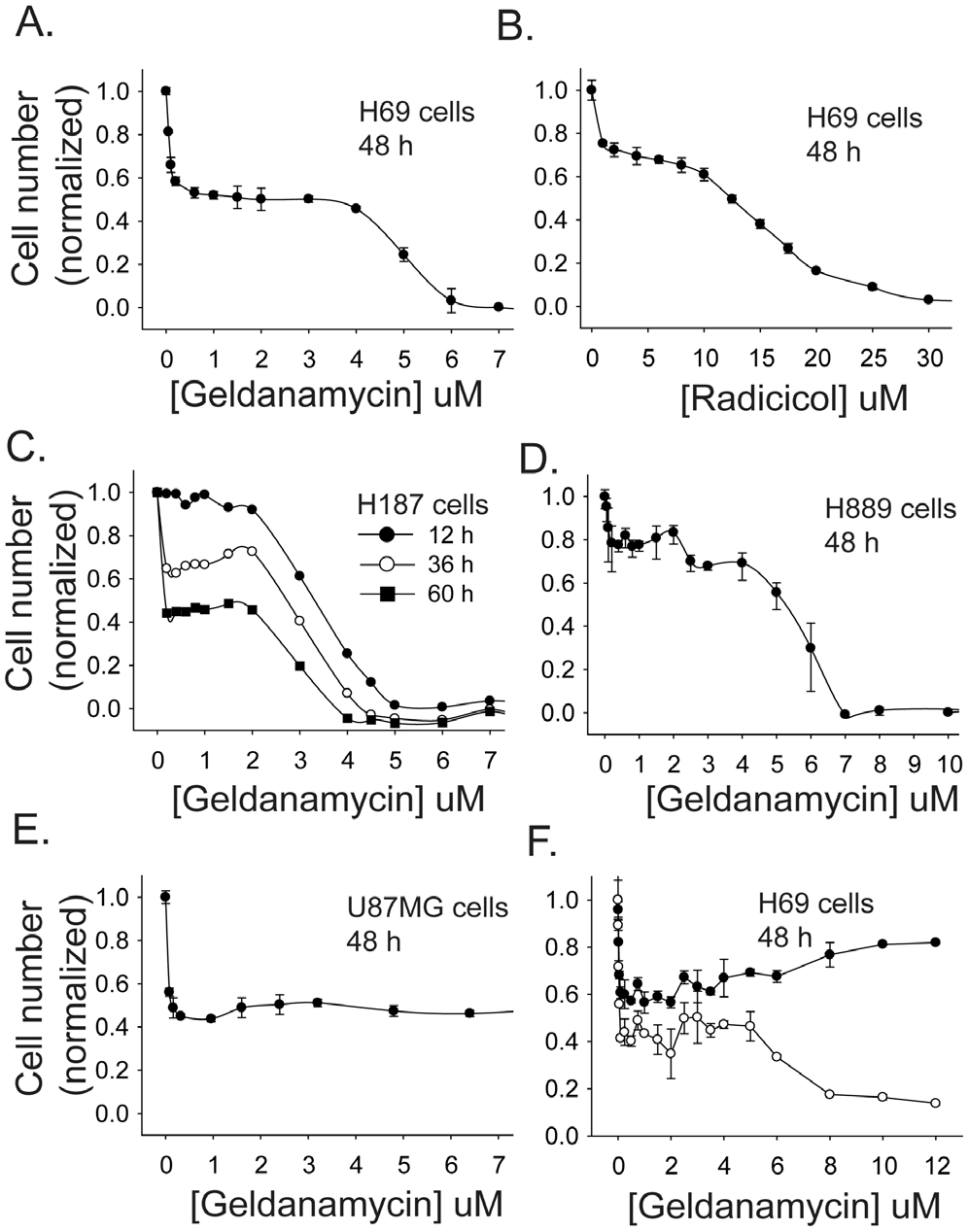
Response of small cell lung cancer cells to Hsp90 inhibition. A, MTT assay of H69 cells treated with geldanamycin for 48 h; B, MTT assay of H69 cells treated with radicicol for 48 h; C, MTT assay of H187 small cell lung cancer cells with geldanamycin for 12, 36 and 60 h; D, MTT assay of H889 cells treated with geldanamycin for 48 h; E, MTT assay of U87MG glioblastoma cells treated with geldanamycin for 48 h; F, H69 cells treated with geldanamycin for 48 h and assayed for viable (○) and total (•) cell counts using a Vi-cell XR cell viability analyzer. Error bars show the mean ± SE of three replicates.

The human small cell lung cancer cell line H69 showed a biphasic response to treatment with a range of geldanamycin concentrations (Figure 1A). In a MTT assay, a 48 h exposure of cells to 0.1 µM geldanamycin decreased viable cell numbers by approximately 40%. Increasing the concentration of geldanamycin to up to 3 µM did not cause a further decrease in viable cell numbers. With concentrations of geldanamycin >4 µM, numbers of viable H69 cells decreased to essentially zero. Radicicol, a second Hsp90 inhibitor that is structurally unrelated to geldanamycin, also gave a biphasic dose response curve with H69 cells, although with a less distinct plateau phase (Figure 1B). Two other small cell lung cancer cell lines (H187 and H889) also show a biphasic response to geldanamycin (Figure 1C and D); however the U87MG glioblastoma cell line showed only the first phase of this response (*i.e*. the phase seen at low geldanamycin concentrations) (Figure 1E).

As the first phase could be potentially due to either inhibition of cell proliferation or selective killing of a subset of SCLC cells, further assays were performed to distinguish between these possibilities. The response of H69 cells to treatment with a range of geldanamycin concentrations was assayed by cell counts, using trypan blue exclusion to distinguish live cells from dead cells (Figure 1F). The live cell counts also showed a biphasic response when assayed by this method. Total cell counts were similar to live cell counts in the first phase of the biphasic response curve but diverged in the second phase. These data show that geldanamycin primarily induces proliferation arrest in H69 cells at low concentrations but induces cell death at high concentrations. The data in Figure 1C, which show MTT assays on H187 cells treated for different periods of time (12, 36 and 60 h) with geldanamycin, are consistent with this conclusion, as the first phase is only evident with longer treatments where there is time for significant cell proliferation to take place in the control. This also shows that the cell death at high geldanamycin concentrations happens relatively rapidly (*i.e.* in less than 12 h).

### Response of H69 cells after withdrawal of Hsp90 inhibition

**Figure 2:**
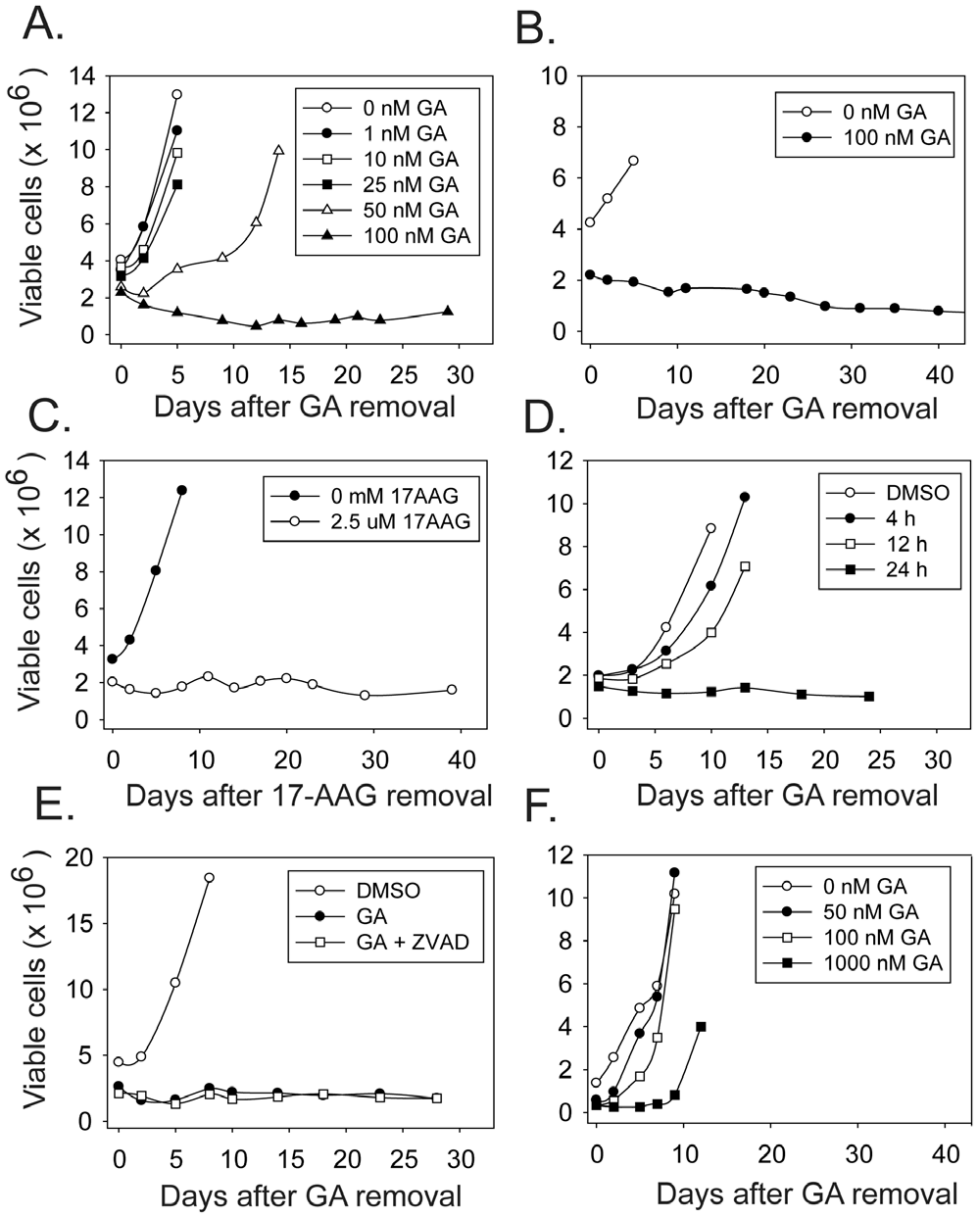
Proliferation after withdrawal of Hsp90 inhibitors. Cells were treated for 48 h with the indicated concentration of inhibitor, which was then removed. Viable cell counts were then determined at the indicated times after inhibitor removal. A, H69 cells treated with geldanamycin; B, H187 cells treated with geldanamycin; C, H69 cells treated with 17-AAG. D, H69 cells treated with 100 nM geldanamycin for 4, 12 or 24 h. E, H69 cells were treated for 48 h with 100 nM geldanamycin in the absence or presence of 100 µM Z-VAD-FMK, a general caspase inhibitor. Z-VAD-FMK treatment was started 1 h before the addition of geldanamycin. F, U87MG cells treated with geldanamycin.

Proliferation arrest induced by drugs may be reversible or irreversible (*i.e.* senescence-like). To distinguish between these possibilities, H69 cells were treated with different concentrations of geldanamycin for two days. Drug was then removed and viable cell counts were monitored. Cell proliferation recovered from treatment with geldanamycin concentrations of 50 nM or less. However, after treatment with 100 nM geldanamycin, a population of viable cells remained that did not increase over a time period of greater than thirty days after removal of drug (Figure 2A). Similar results were obtained with the H187 and H889 small cell lung cancer cell lines (Figure 2B and Figure S1A) and in H69 cells treated with the clinically-relevant Hsp90 inhibitor 17-AAG (Figure 2C). H69 cells required a minimum of 24 h of exposure to geldanamycin to induce irreversible proliferation arrest (Figure 2D) and the induction of irreversible proliferation arrest was unaffected by caspase inhibition (Figure 2E). For comparison the same experiment was performed on the U87MG human glioblastoma cell line (Figure 2F). These cells recover growth after treatment with 100 nM geldanamycin and also recover from a treatment with a ten-fold higher concentration of geldanamycin. The sustained proliferation arrest induced by geldanamycin is therefore not a universal response of cancer cells but rather is cancer cell- type specific.

**Figure 3:**
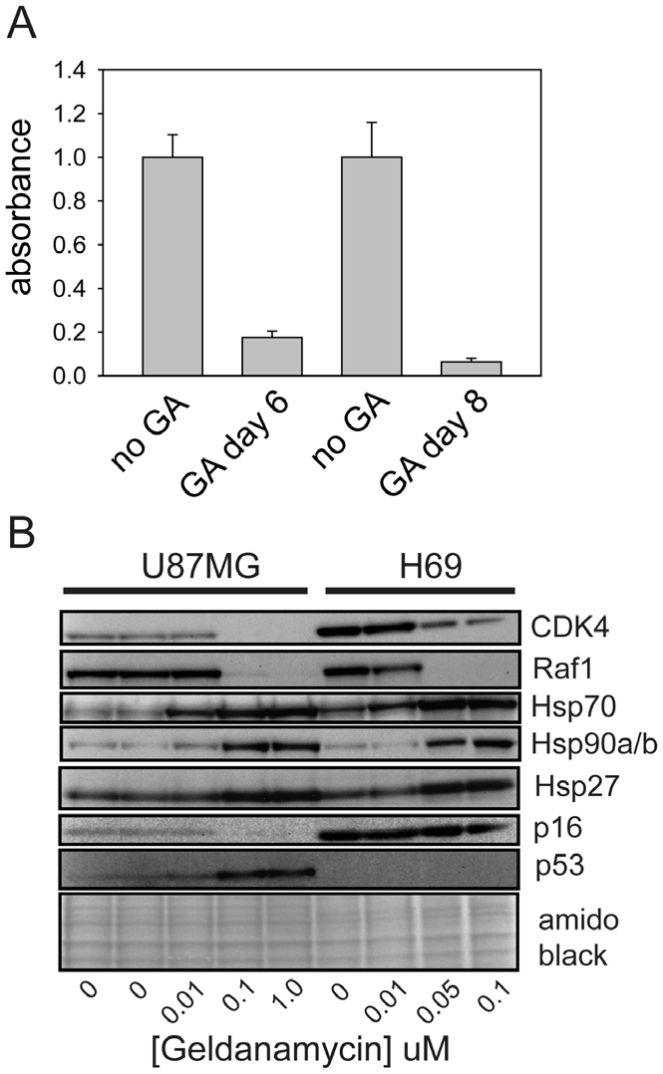
BrdU incorporation and client protein responses in Hsp90 inhibitor-treated cells. A. H69 cells were treated for 48 h with 100 nM geldanamycin. Geldanamycin was then removed. Six or eight days after drug removal, 25,000 treated cells (GA) per well were plated in ninety-six well plates along with the same number of untreated H69 cells for comparison (no GA). Cells were allowed to uptake BrdU for 16 h and BrdU incorporation was then assayed as described in Materials and Methods. Results were normalized to the values for untreated cells. Data shown are the mean ± SD of six replicates. B. Cells were treated for 48 h with geldanamycin. Total cell lysates were then collected and analyzed by Western blotting using antibodies to the indicated proteins. The bottom panel shows a blot stained for total protein with amido black prior to antibody incubation. Equal amounts of DMSO were added to cells for each treatment, except in the leftmost lane, where DMSO was omitted.

To confirm the sustained growth arrest by a second method, incorporation of BrdU into H69 cells that had been exposed to 100 nM geldanamycin was also assessed. Consistent with viable cell counts, this assay showed that H69 cells failed to recover their proliferative capacity after geldanamycin removal (Figure 3A).

### Changes in Hsp90 client proteins and heat shock proteins in response to Hsp90 inhibition

The effects of different concentrations of geldanamycin on known Hsp90 clients were also assessed in H69 cells (Figure 3B, right four lanes of blot). Substantial depletion of the Hsp90 client proteins Cdk4 and Raf1 were seen at geldanamycin concentrations as low as 50 nM. A second well-described response of cells to Hsp90 inhibition is the induction of other heat shock proteins that occurs via activation of Hsf1 [19]. As with client protein responses, geldanamycin concentrations as low as 50 nM caused increases in Hsp70, Hsp27 and Hsp90α/β. These geldanamycin concentrations parallel those that cause proliferation arrest, but are about 100 times less than the concentrations that cause substantial cell death.

U87MG and H69 cells showed very similar sensitivities to geldanamycin with respect to client protein decreases and heat shock protein increases (Figure 3B). This indicates that the difference in behavior between H69 and U87MG cells after removal of geldanamycin is not a consequence of differences in drug metabolism (*e.g.* multidrug transporter activity or diaphorase activity, both of which affect geldanamycin activity [20], [21]). Rather it appears that the different responses of these cells after withdrawal of geldanamycin are due to differences that are downstream of Hsp90 inhibition. Western blot analysis confirmed that H69 cells lacked detectable p53 (Figure 3B), in agreement with Smith *et al*., who showed that there is a stop mutation in *P53* exon 5 in this cell line [22]. p16Ink4a was present in H69 cells and its levels were unaffected by treatment with geldanamycin for 48 h (Figure 3B).

### Senescence markers in growth-arrested small cell lung cancer cells

**Figure 4:**
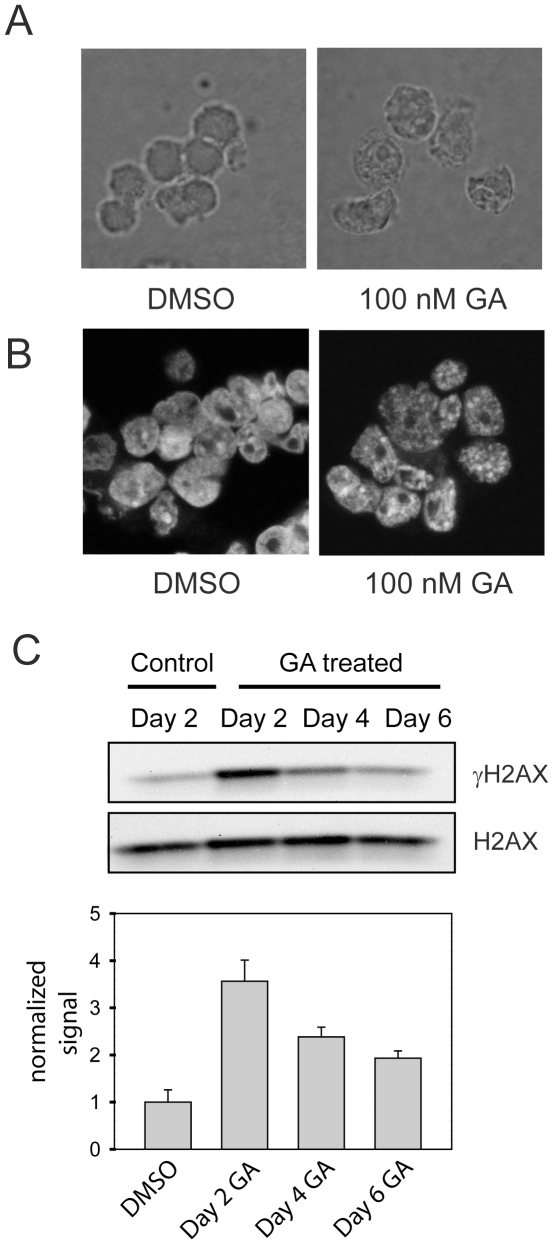
Senescence markers in Hsp90-inhibitor-treated small cell lung cancer cells. A. H69 cells were treated with DMSO alone or 100 nM geldanamycin for 48 h. Geldanamycin was then removed. Eight days later cells were allowed to settle onto poly-L-lysine-coated coverslips, fixed with paraformaldehyde and photographed under phase contrast using a 63X objective lens. B. SAHF formation after treatment of cells with Hsp90 inhibitors. H69 cells were treated with DMSO or 100 nM geldanamycin for 48 h. Cells were then fixed, stained with DAPI and examined by fluorescence microscopy. C. H69 cells were treated with DMSO (control) or 100 nM geldanamycin. DMSO and geldnamycin were then removed on Day 0. Total cell lysates were collected on the indicated days after geldanamycin removal and analyzed for γH2AX and H2AX levels by Western blotting. The graph shows densitometry for Western blot analyses of three biological replicates. Data were normalized to the vehicle-treated control cells and are shown as the mean ± SE.

The irreversible proliferation arrest induced by Hsp90 inhibition in small cell lung cancer cells suggested that these cells had become senescent. To assess this further, additional markers of senescence were assessed. Figure 4A shows the morphology of suspension cultures of untreated H69 cells eight days after geldanamycin removal. Treated cells show some enlargement and increased cytoplasmic granularity, characteristic features of senescent cells. Another common feature of senescent cells is the presence of senescence-associated heterochromatin foci (SAHF) [23]. Consistent with senescence induction, inhibition of Hsp90 with geldanamycin led to the formation of SAHF in H69 cells as demonstrated by DAPI staining (Figure 4B). SAHF were maintained after removal of geldanamycin, as expected for a senescence marker and were enriched in histone H3 trimethylated on lysine 9, consistent with previous studies (Figure S1B) [24]. Activation of a DNA damage response is an important feature of both replicative and premature senescence that contributes to the growth arrest phenotype of senescent cells [25]. A central feature of the DNA damage response is the generation of phosphorylated histone H2AX (γH2AX) at sites adjacent to DNA damage. Both geldanamycin and radicicol treatments increased levels of γH2AX in H69 cells (Figure S1C). γH2AX levels remained elevated for up to six days after geldanamycin removal, the longest time point assessed (Figure 4C). This is consistent with reports that senescent cells maintain an activated DNA damage response for an extended period after the induction of senescence [25], [26].

### Isolation of variant H69 cells that bypass geldanamycin-induced senescence

**Figure 5:**
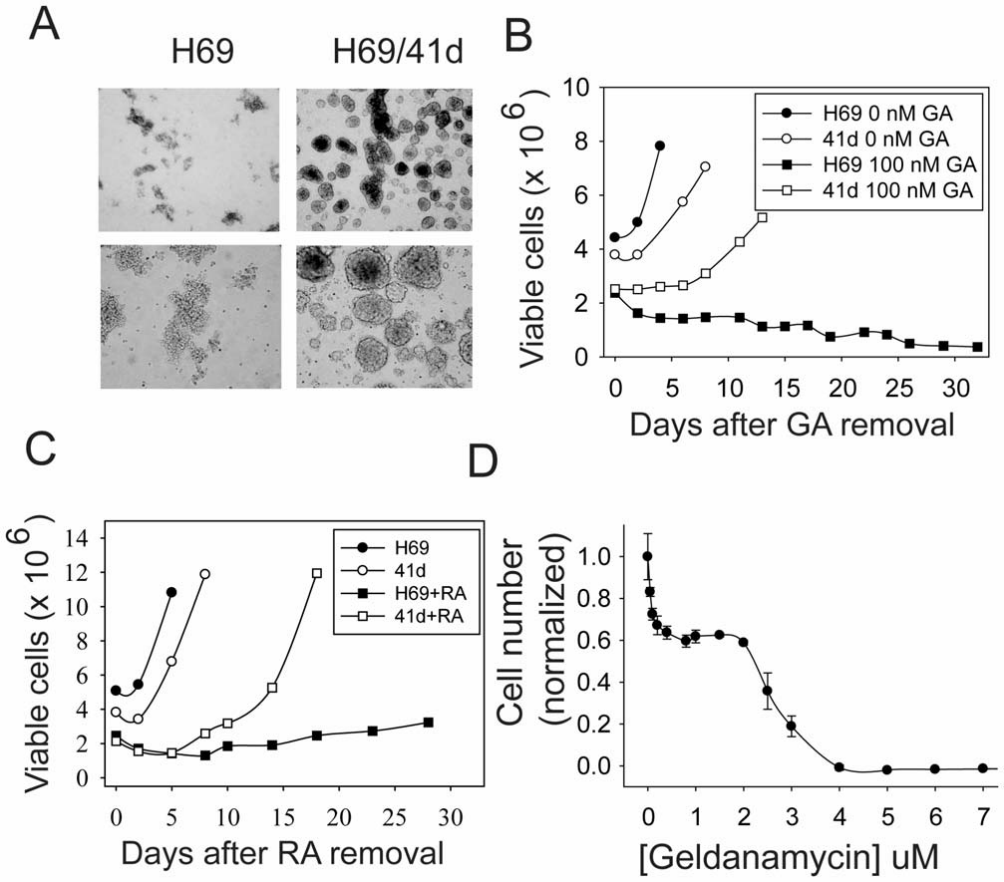
Properties of variant H69 cells that recover growth after Hsp90 inhibitor withdrawal. A. Photographs of H69 and H69/41d cells under phase microscopy, at low (top panels) and high (bottom panels) magnification; B. H69 and H69/41d cells were treated with 100 nM geldanamycin for 48 h. Geldanamycin was then removed and numbers of viable cells were assessed at the indicated times after drug removal. C. H69 and H69/41d cells were treated with 5 µM radicicol for 48 h. Radicicol was then removed and numbers of viable cells were assessed at the indicated times after drug removal. D. MTT assay of H69/41d cells treated for 48 h with geldanamycin.

**Figure 6:**
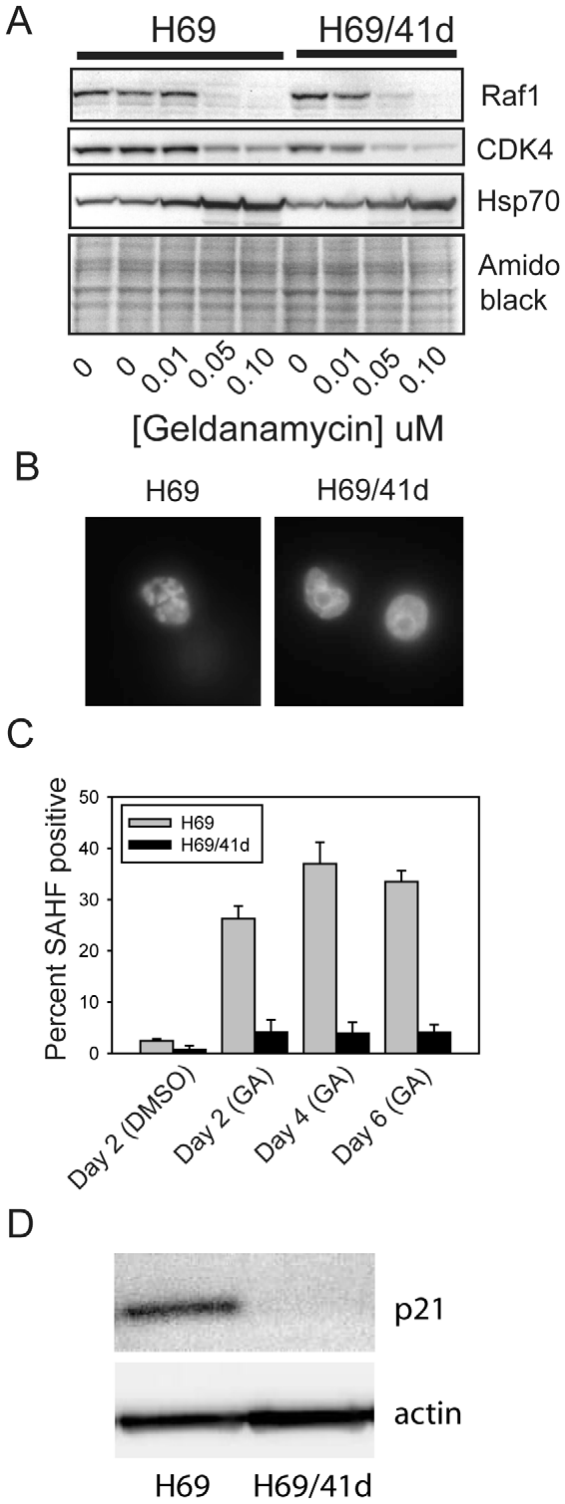
Client protein degradation and SAHF induction in H69/41d cells. H69 and H69/41d cells were treated for 48 h with geldanamycin or DMSO as vehicle control. A. Total cell lysates were collected after 48 h treatment and analyzed by Western blotting as in Figure 3. B. DMSO-treated cells were stained for SAHF 2 days after removing the DMSO. Geldanamycin-treated cells were stained for SAHF 2, 4 and 6 days after drug removal. Representative fluorescence microscopy images of H69 and H69/41d cell nuclei, six days after removal of geldanamycin, are shown. C. SAHF in H69 (light gray) and H69/41d cells (dark gray) were counted at the indicated times after drug removal. Data are expressed as the percent of nuclei that are SAHF positive. Bars show the mean percentage ± SE. D. Total cell extracts of untreated H69 and H69/41d cells were analyzed for expression of p21 protein by Western blotting.

While H69 cells maintained a proliferation-arrested state for more than thirty days after removal of geldanamycin, after approximately forty days a population of cells did start to proliferate in one experiment. We have designated this population as H69/41d cells. H69/41d cells have a distinct morphology: while H69 cells are non-adherent cells that form ragged aggregates, the variant cells, while also non-adherent, form tight spheres reminiscent of those seen with stem cell populations (Figure 5A). Upon retesting, these cells still underwent proliferation arrest in response to 100 nM geldanamycin, but were able to recover proliferative capacity upon removal of drug, similar to what we observed in glioblastoma cells (Figure 5B). H69/41d cells were also able to recover proliferative capacity after treatment with the Hsp90 inhibitor radicicol (Figure 5C). Compared to the parent H69 cell line, H69/41d cells showed similar responses to Hsp90 inhibition with respect to proliferation inhibition in the presence of drug, although cell death tended to occur at slightly lower concentrations than seen with H69 cells (Figure 5D). Degradation of client proteins and induction of other heat shock proteins was also similar in the two cell lines (Figure 6A). As above, this rules out differences in drug processing between the two cell populations, and indicates that the differences are due to events downstream of Hsp90 inhibition that specifically affect whether cells undergo reversible or irreversible proliferation arrest. SAHF formation was also impaired in H69/41d cells (Figure 6B). In H69 cells, SAHF were detected maximally in approximately 35% of cells (Figure 6C). In contrast, geldanamycin treatment only caused a modest increase in SAHF in H69/41d cells and SAHF were detected in 4.5% of cells maximally (Figure 6C).

To directly assess the genetic relationship of H69 cells with H69/41d cells, DNA was isolated from both cell lines and analyzed using Affymetrix Genome-Wide Human SNP Array 6.0 chips, which contain probes for 900,000 single nucleotide polymorphism and a similar number of probes to assess copy number variation. Figure S2 shows karyotypes of the two cell lines reconstructed from this data (each is compared to an Affymetrix reference karyotype). H69 cells show extensive chromosomal aberrations as expected for cancer cells. These losses, gains and regions of amplification/copy number variation are very similar between H69 and H69/41d cells, showing unequivocally that they are clonally related. Some differences were evident between the two cell lines. These include a 4.8 Mbp deletion on the single copy of chromosome 2 that contains approximately nineteen genes and a 9.6 Mbp deletion on one copy of chromosome 11 that contains approximately 83 genes. There is also a gain of the long arm of chromosome 3 and a loss of a partial long arm of chromosome 15. However, the SNP/copy number analysis did not point to a single defined genetic locus that might explain why H69/41d cells are able to evade Hsp90 inhibitor-induced senescence. As a second approach to determine the mechanism by which these cells evade Hsp90 inhibitor-induced senescence, cells were screened for a number of proteins that have previously been shown to have important roles in senescence. p21 (CDKN1A, p21Waf1/Cip1) is one such protein that is of particular interest as it has been shown to induce senescence in cancer cells lacking p53 [27]. p21 is expressed in H69 cells but is undetectable in H69/41d cells (Figure 6D). Given its known role in the induction and maintenance of senescence [26], the loss of p21 expression provides a plausible explanation for the failure of H69/41d cells to undergo senescence in response to Hsp90 inhibitors.

## Discussion

In previous studies, the most commonly described response of cancer cells to Hsp90 inhibition has been cell cycle arrest. This cell cycle arrest has generally been assumed to be reversible in nature, although most studies did not address this directly. Growth arrest can occur at either the G1/S or G2/M transition [28], [29] and is likely to be a consequence of the Hsp90 dependence of proteins such as CDK4, cdc2 and polo-like kinase [30]-[32]. In a subset of cancer cell types, Hsp90 inhibitors have been shown to induce apoptosis. These include small cell lung cancer cells and multiple myeloma cells [18], [33].

Aside from transient cell cycle arrest and cell death, a third cellular fate for cancer cells is senescence. For cancer cells, this is sometimes referred to as “premature” or “accelerated” cellular senescence to distinguish it from the replicative senescence that normal cells undergo upon reaching their Hayflick limit [34], [35]. Senescence in cancer has been studied in two contexts: the first of these is the senescence seen in response to oncogene activation, where it may play a role in protecting organisms from developing cancer; the second of these is as a response to cancer therapeutics [36]. Chemotherapy agents that damage DNA appear to induce senescence to a much greater extent than agents that target microtubules [37]. This is more likely to occur with exposure to lower doses of drug, with higher doses causing apoptosis. Chemotherapy-induced senescence has been observed both in cell culture and in animal models [38].

The key, defining features of senescent cells are that they remain metabolically active but undergo a sustained withdrawal from the cell cycle that is not reversed by standard mitogenic stimuli. After transient exposure to low concentrations of geldanamycin, small cell lung cancer cells remained alive and metabolically active (as indicated by trypan blue exclusion and MTT assays). However these cells underwent a proliferation arrest that was sustained for greater than thirty days, in spite of the regular addition of fresh media containing fetal calf serum, a rich source of mitogens. The proliferation arrest was evident both from live cell counts and from BrdU incorporation assays.

In addition to permanent proliferation arrest, additional markers of senescence have been developed, although none of these is currently seen as being a definitive marker of the senescent state [39]. A characteristic set of morphological changes have been described for senescent cells that include cell enlargement and an increased granularity of the cytoplasm [40]. These features were evident in H69 small cell lung cancer cells in which sustained proliferation arrest had been induced by Hsp90 inhibitors. (They do not show the flattening described for adherent cells, as the small cell lung cancer cells used in this study grow in suspension.) SAHF are another marker that is commonly observed in senescent cells and that are thought to have a role in maintaining the senescent phenotype. SAHF were present in small cell lung cancer cells in which sustained proliferation arrest had been induced by Hsp90 inhibitors. Expression of SAHF was maintained for up to six days after removal of Hsp90 inhibitor (the longest time point examined) and the time course for their appearance was similar to previous studies on the induction of premature senescence by various agents [41], [42]. Activation of the DNA damage response is also commonly observed in senescence, where it has a role in both the initiation and maintenance of the senescent phenotype [25], [26]. Hsp90 inhibitors (both geldanamycin and radicicol) activated a DNA damage response that was maintained after inhibitor removal. This finding is also consistent with a senescence phenotype and provides a mechanism by which these inhibitors activate senescence.

DNA damage, either telomeric or non-telomeric, is the most widely characterized inducer of senescence. As well as being a marker of senescence, the activation of the DNA damage response by Hsp90 inhibitors also provides a mechanism by which they induce senescence in small cell lung cancer cells. Previous studies have also pointed to a link between Hsp90 and DNA damage: both telomerase and the Fanconi anemia DNA damage response pathway are dependent on Hsp90 for their activity [43], [44].

Senescence-associated β-galactosidase (SAβgal) has also been widely used as a senescence marker [35]. SAβgal activity is detected as a consequence of the expansion of the lysosomal compartment in senescent cells, but is not required for senescence induction or maintenance [45]. Small cell lung cancer cells treated with Hsp90 inhibitors were not positive for SAβgal. Adriamycin also did not induce SAβgal in small cell lung cancer cells when used at concentrations that induced this marker in other cancer cell types. This suggests that SAβgal may not be a useful marker of senescence in small cell lung cancer. A possible explanation is that these cells have scant cytoplasm and are specialized secretory cells that may have a relatively small lysosomal compartment.

Taken together, the above data show that Hsp90 inhibitors induce a sustained proliferation arrest that has features consistent with premature senescence. This premature senescence occurred at concentrations of geldanamycin that correlated closely with the concentrations required to induce the degradation of well-established Hsp90 client proteins and to induce the expression of other heat shock proteins (a widely used marker of Hsp90 inhibition). Premature senescence was also induced by 17-AAG (tanespimycin; 17-allylamino-17-demethoxygeldanamycin), a geldanamycin derivative that has been tested in clinical trials [46]. 17-AAG induced senescence at a concentration that is similar to the concentrations achieved in the plasma of patients treated at the maximum tolerated dose of this compound [12], suggesting that this response is clinically relevant. Senescence induction by Hsp90 inhibitors requires neither p53 nor Rb, as both of these tumor suppressors are known to be mutated in the H69 cell line used in this study.

In some experiments, populations of cells did start to grow after long term culture of senescent H69 cells. One of these cell populations, designated H69/41d, was characterized in detail. SNP and copy number analysis of these cells shows that while they are clearly derived from H69 cells they have a distinct set of genetic changes. These cells show similar responses to Hsp90 inhibition as the parent H69 cells with respect to proliferation inhibition in the presence of drug and induction of cell death at high geldanamycin concentrations. However they undergo a reversible, rather than irreversible proliferation arrest in response to Hsp90 inhibition. H69/41d cells also show identical responses to the parent cell line with respect to degradation of Hsp90 client proteins and induction of other heat shock proteins. Thus this form of “resistance” to Hsp90 inhibition is clearly distinct from that described in previous studies, where resistance was due to alterations in drug metabolizing enzymes and was reflected in a requirement for higher doses to see cellular effects [47]. The isolation of these cells demonstrates that, in principle, small cell lung cancer cells with additional genetic alterations can evade Hsp90-induced premature senescence. p21 (CDKN1A) has previously been shown to be an important regulator of senescence, a property that appears to be independent of its inhibitory activity against cyclin-dependent kinases [48]. p21 is regulated by both p53 and p53-independent mechanisms and can induce sensescence in cells that lack p53 (as is the case for H69 cells) [27]. The absence of p21 in H69/41d cells provides a mechanism by which these cells evade premature senescence in response to Hsp90 inhibition.

We observed two distinct responses to Hsp90 inhibitors in small cell lung cancer cells, the first being the premature senescence described above and the second being cell death, which is seen at much higher concentrations of Hsp90 inhibitors. The two responses are temporally distinct, in that cell death is maximal after only 12 h of exposure to Hsp90 inhibitor, while premature senescence requires a minimum of 24 h exposure to drug. These two responses were reflected in a biphasic dose response curve in MTT assays, which was very distinct with geldanamycin but was more subtle with radicicol (see below). The biphasic response is only evident when a detailed analysis of the dose effects of Hsp90 inhibitors at low concentrations is performed and when long treatments with drug are used, where there is sufficient time for cell proliferation to occur in untreated samples. The induction of cell death that we observed is consistent with previous observations, where it was shown that Hsp90 inhibitors activate the intrinsic pathway of apoptosis in small cell lung cancer cells [18]. However we find that the concentration of geldanamycin required to induce cell death is approximately 100 times higher than that required to inhibit cytosolic Hsp90, as assessed by degradation of Hsp90 client proteins and induction of heat shock proteins. The cell death seen in small cell lung cancer cells is therefore due to inhibition of a target other than cytosolic Hsp90; likely alternate targets include either the endoplasmic reticulum Hsp90 paralog Grp94 or the mitochondrial Hsp90 paralog Trap1. Geldanamycin binds to purified Grp94 and Trap1 with dissociation constants in the 1-2 µM range [8], [10]; the IC_50_ for the second phase of the curve in our geldanamycin MTT assays is approximately 5 µM, consistent with either Grp94 or Trap1 as alternate targets. The large difference in the affinity of geldanamycin for cytosolic Hsp90 in cancer cells (low nanomolar range) and either Grp94 or Trap1 may explain why a biphasic dose response is clearly seen with this compound in MTT assays; for compounds where the affinities are relatively close, this would not be observed as readily. In the previous report linking small cell lung cancer cell apoptosis to inhibition of cytosolic Hsp90, the Grp94 and Trap1 binding affinities of the compounds used were not assessed [18]. Previous work has indicated that inhibition of Trap1 (along with mitochondrial Hsp90) is cytotoxic to cancer cells [49], [50]. Similarly, inhibition of Grp94 can also induce cell death under some conditions [51]. Trap1 and Grp94 are therefore candidate alternate targets for the effects of Hsp90 inhibitors on small cell lung cancer cells both on the basis of their known affinities for Hsp90 inhibitors and their established roles in the prevention of cell death.

Our studies support the earlier conclusion that small cell lung cancer may be a candidate for treatment with Hsp90 inhibitors [18]. However we find that a senescence-like proliferation arrest is the primary response of these cells to concentrations of Hsp90 inhibitors that inhibit cytosolic Hsp90. This suggests that disease stabilization may be a more likely outcome in patients rather than tumor regression, and that small populations of cancer cells with additional genetic alterations may be able to escape senescence induction. If, as our data suggests, the apoptotic effect of Hsp90 inhibitors in small cell lung cancer is due to inhibition of other Hsp90 family members, such as Grp94 or Trap1, it will be important to develop new drugs that are optimized for selectivity and potency for these targets.

## Materials and Methods

### Chemicals and antibodies

Geldanamycin and radicicol were from Sigma-Aldrich Canada Ltd. (Oakville, ON, Canada). 17-AAG (17-(Allylamino)-17-demethoxygeldanamycin) and the caspase inhibitor Z-VAD-FMK were from Calbiochem (San Diego, CA, USA). MTT reagent (3-(4,5-dimethylthiazol-2-yl)-2,5-diphenyl tetrazolium bromide) was from Sigma-Aldrich Canada Ltd. (Oakville, ON, Canada). Cdk4, p21 and Hsp70 mouse monoclonal antibodies were from Neomarkers (Fremont, CA, USA). Raf1 rabbit polyclonal antibody was from Santa Cruz Biotechnology Inc. (Santa Cruz, CA, USA). Hsp90α/β mouse monoclonal and Hsp27 rabbit polyclonal antibodies were from Stressgen (Ann Arbor, MI, USA). Actin and p16 mouse monoclonal antibodies were from Sigma-Aldrich Canada Ltd. (Oakville, ON, Canada). Antibodies to γH2AX (phospho-Histone H2A.X (Ser139)) and histone H2A.X were from Millipore (Billerica, MA, USA). Rabbit polyclonal antibody to histone H3 tri-methylated K9 was from Abcam (Cambridge, MA, USA). p53 mouse monoclonal antibody was from Oncogene Science (Uniondale, NY, USA).

### Cell lines

Human H69, H187 and H889 small cell lung cancer cell lines were from American Type Culture Collection (Manassas, VA, USA). All cell lines were passaged for less than six months continuously and were routinely checked for mycoplasma contamination. Small cell lung cancer cell lines were grown as suspension cultures in RPMI-1640 medium containing 25 mM HEPES and L-glutamine (Invitrogen, Carlsbad, CA, USA) supplemented with 100 units/ml penicillin, 100 µg/ml streptomycin, 1 mM sodium pyruvate and 10% (v/v) fetal bovine serum. The human U87MG glioblastoma cell line was obtained from Dr. W. Cavenee (Ludwig Institute for Cancer Research, La Jolla, CA, USA) and was cultured in Dulbecco's modified Eagle's medium (DMEM) supplemented with 100 units/ml penicillin, 100 µg/ml streptomycin, 2 mM glutamine and 10% (v/v) of a 2∶1 mixture of donor bovine serum and fetal bovine serum. All cell lines were cultured at 37°C and 5% CO_2_.

### Cell counts

Total and viable cell numbers were measured using a Vi-cell XR cell viability analyzer from Beckman Coulter Canada Inc. (Mississauga, ON, Canada).

### Growth assays

Cells were added to 5 ml of media in T25 tissue culture flasks and 24 h later were treated with drug or the drug vehicle DMSO (dimethyl sulfoxide) for the specified time. Cells were pelleted by centrifugation, media with drug was removed, and cells were washed with sterile PBS and centrifuged again. Cells were then added to new flasks with fresh media (day 0). Aliquots were counted for total and viable cell numbers on the specified days and fresh media was added to replenish and maintain the total volume of media in the flask. Cell proliferation was also assessed using the BrdU Cell Proliferation Assay kit from Calbiochem (San Diego, CA, USA).

### MTT assays

Cells were added to 100 µl media in 96-well tissue culture plates and 24 h later were treated with drug or the drug vehicle DMSO for the specified time. 25 µl of MTT reagent was added (5 mg/ml in sterile PBS) and incubated for 24 h at 37°C and 5% CO_2_. The cells were lysed and the formazan product was dissolved for 30 min with 100 µl of lysis detergent (20% SDS (BioShop Canada Inc., Burlington, ON, Canada) and 40% DMF (VWR International, Mississauga, ON, Canada). The absorbance was measured at 570 nm using a Dynex MRX microplate reader (Dynex Technologies, Chantilly, VA, USA).

### Western blot analysis

Western blotting was performed as described previously [52]. To ensure equal loading and transfer, blots were stained with amido black prior to blocking and incubation with primary antibody.

### SAHF staining

H69 cells were centrifuged, washed twice with cold PBS for 5 min, fixed in 4% paraformaldehyde at room temperature for 30 min, and washed twice with PBS for 5 min. Cells were stained for 4 min with 0.13 µg/µl DAPI (4′,6-diamidino-2-phenylindole, Invitrogen, Carlsbad, CA, USA). Cells were then washed twice with PBS for 5 min and mounted on slides in 5 µl PBS and 5 µl of ProLong Gold mounting medium (Invitrogen, Carlsbad, CA, USA). Fluorescence microscopy was performed using an AxioSkop 2 MOT microscope with a Zeiss FluoArc mercury lamp and Zeiss AxioVision release 3.1 software (Carl Zeiss Canada Ltd., Toronto, ON, Canada). Percentages of cells containing SAHFs were determined by counting total and SAHF- containing nuclei under a 100X objective. A minimum of three randomly-chosen fields containing a minimum total of 100 nuclei were scored independently by two reviewers and averaged. For detection of histone H3 trimethylated on lysine 9, H69 cells were allowed to settle onto cover slips coated with 0.1% poly-L-lysine (Sigma-Aldrich, St. Louis MO) and fixed with paraformaldehyde as above. Immunofluorescence was then performed as described previously using anti-histone H3 tri-methylated K9 antibody at a 1∶5000 dilution [5].

### Microarray-based DNA analysis

DNA was isolated using the DNeasy Blood and Tissue kit from Qiagen (Mississauga, ON, Canada). Analysis using Genome-Wide Human SNP Array 6.0 chips from Affymetrix Inc. (Santa Clara, CA, USA) was performed at the Centre for Applied Genomics at the Hospital for Sick Children, Toronto, ON, Canada.

Data analysis was performed using the Genotyping Console software version 3.0.1 from Affymetrix Inc. (Santa Clara, CA, USA).

## Supporting Information

Figure S1 A. H889 human small lung cancer cells were treated with 100 nM geldanamycin for 48 h. Viable cell numbers were determined at the indicated days after geldanamycin removal as indicated in Figure 2. B. H69 were treated with 100 nM geldanamycin for 48 h. Four days after removal of the drug, cells were allowed to settle onto poly-L-lysine-coated coverslips and fixed with paraformaldehyde. Histone H3 trimethylated on lysine 9 was then detected by immunofluorescence (red) with counterstaining of nuclei using DAPI (green).
(1.16 MB TIF)

Figure S2 Karyotypes of H69 and H69/41d cells. DNA was isolated from H69 and H69/41d cells and analyzed using Affymetrix Genome-Wide Human SNP Array 6.0 chips. The labels above each column indicate the chromosomes depicted by each small panel (e.g. column one has five small panels that show data for chromosomes one, at the top of the column, through five, at the bottom). Each small panel shows copy number on a scale of one to five for H69 cells (in blue) and H69/41d cells (in green) for one chromosome, displayed using the smoothsignal display option in Affymetrix Genotyping Console software. Data files are available from the authors on request.
(0.66 MB TIF)
