# Supplementary figures and images for: Induction of Premature Senescence by Hsp90 Inhibition in Small Cell Lung Cancer
Source: PLoS One. 2010 Jun 11;5(6):e11076. doi: 10.1371/journal.pone.0011076 (PMC2884022; doi:10.1371/journal.pone.0011076)

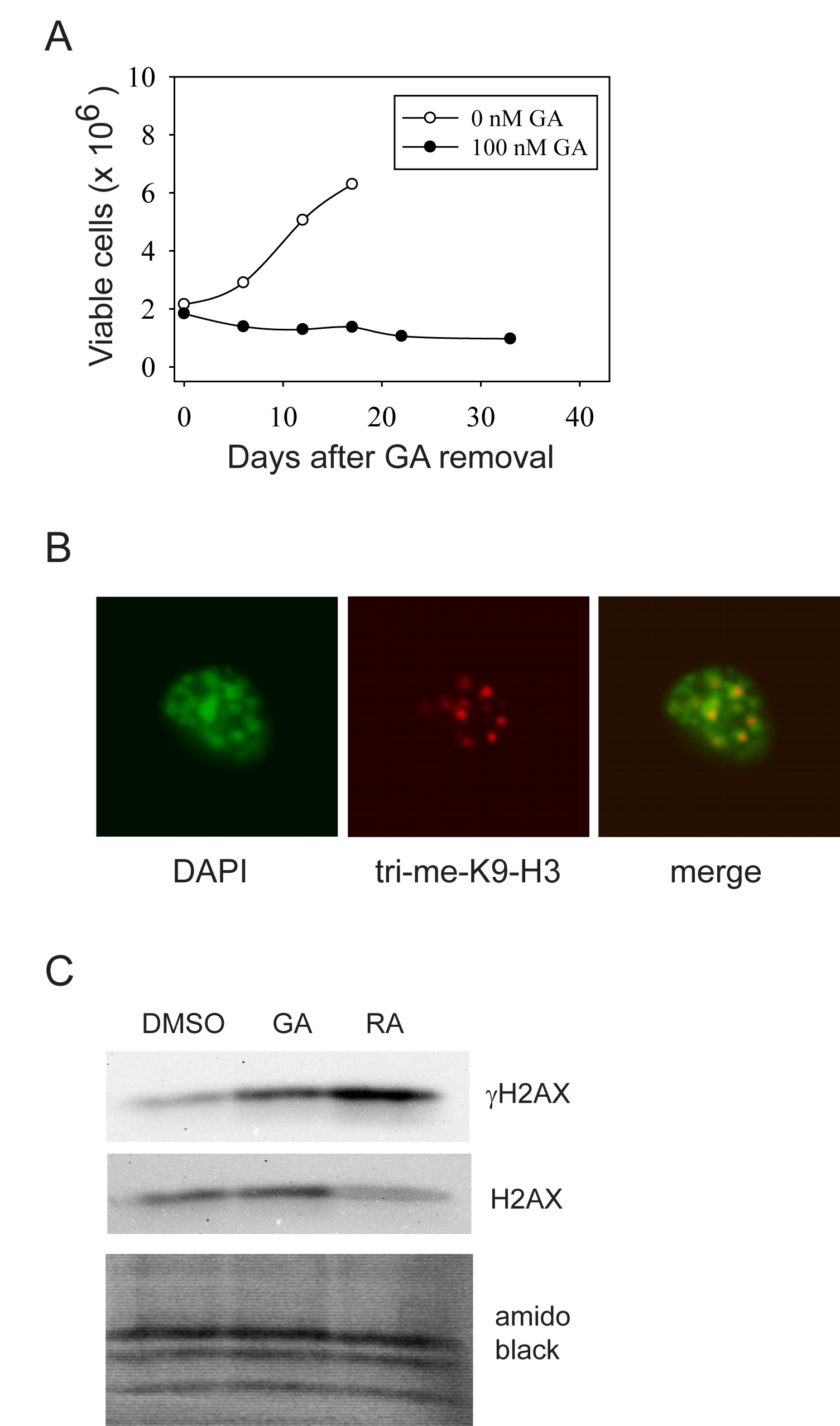

Supplement: Figure S1 — A. H889 human small lung cancer cells were treated with 100 nM geldanamycin for 48 h. Viable cell numbers were determined at the indicated days after geldanamycin removal as indicated in Figure 2. B. H69 were treated with 100 nM geldanamycin for 48 h. Four days after removal of the drug, cells were allowed to settle onto poly-L-lysine-coated coverslips and fixed with paraformaldehyde. Histone H3 trimethylated on lysine 9 was then detected by immunofluorescence (red) with counterstaining of nuclei using DAPI (green). (1.16 MB TIF) [file pone.0011076.s001.tif]

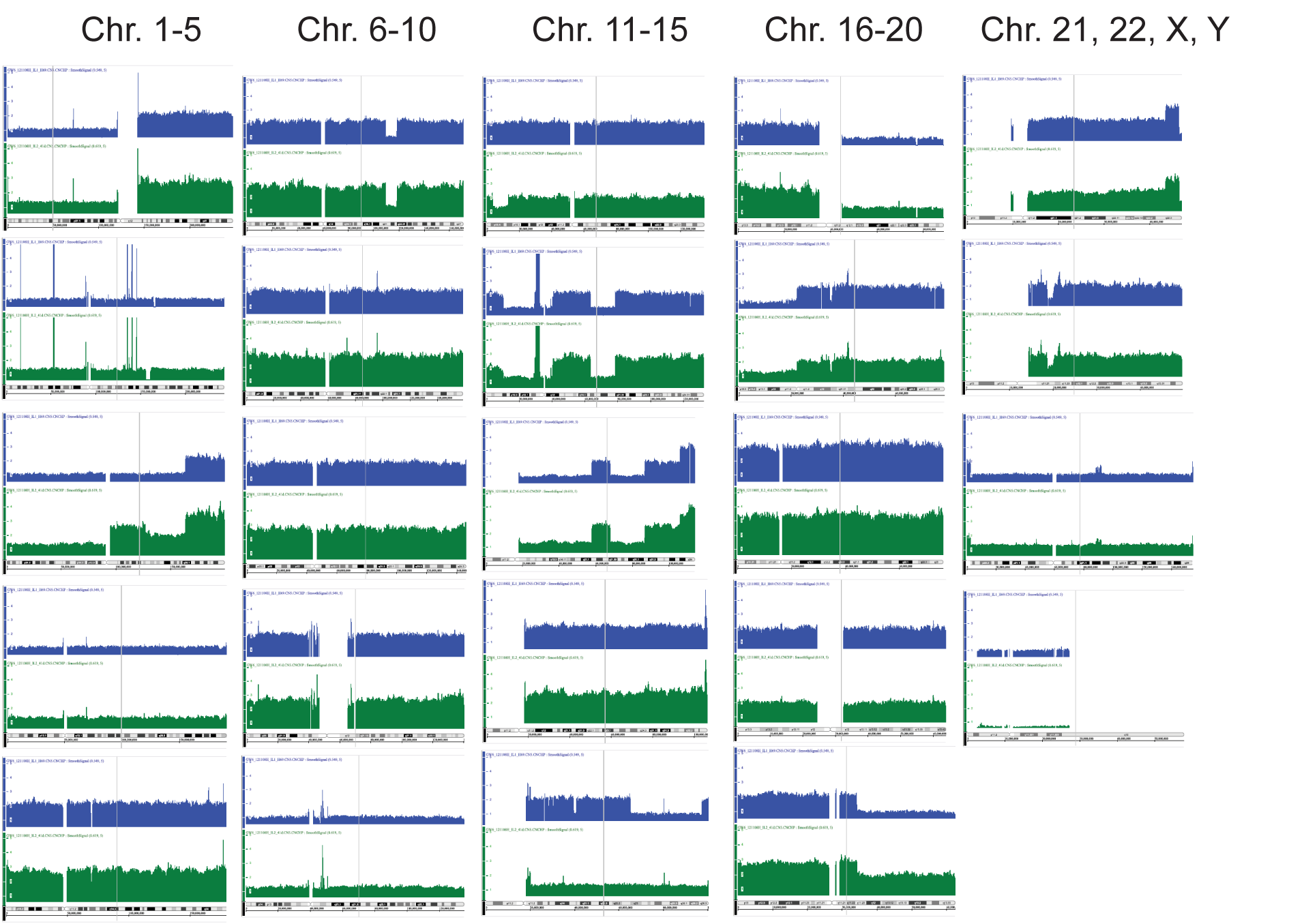

Supplement: Figure S2 — Karyotypes of H69 and H69/41d cells. DNA was isolated from H69 and H69/41d cells and analyzed using Affymetrix Genome-Wide Human SNP Array 6.0 chips. The labels above each column indicate the chromosomes depicted by each small panel (e.g. column one has five small panels that show data for chromosomes one, at the top of the column, through five, at the bottom). Each small panel shows copy number on a scale of one to five for H69 cells (in blue) and H69/41d cells (in green) for one chromosome, displayed using the smoothsignal display option in Affymetrix Genotyping Console software. Data files are available from the authors on request. (0.66 MB TIF) [file pone.0011076.s002.tif]
